# Vitreous MMP-2, TIMP-1, and TIMP-2 Levels in Vitreoretinal Pathologies: A Prospective Analysis of 181 Eyes

**DOI:** 10.3390/ijms26188947

**Published:** 2025-09-14

**Authors:** Rami Al-Dwairi, Tamam El-Elimat, Abdelwahab Aleshawi, Ahmed Al Sharie, Seren Al Beiruti, Abdallah Sharayah, Mohammad Al Qudah, Laith Abu zreig, Walaa Awad, Hosni Alzoubi

**Affiliations:** 1 Division of Ophthalmology, Department of Special Surgery, Faculty of Medicine, Jordan University of Science and Technology, Irbid 22110, Jordan; 2 Department of Medicinal Chemistry and Pharmacognosy, Faculty of Pharmacy, Jordan University of Science and Technology, Irbid 22110, Jordan; 3 Department of Internal Medicine, Southeast Health, Dothan, AL 36301, USA

**Keywords:** rhegmatogenous retinal detachment, vitreous, TIMP2, pars plana vitrectomy

## Abstract

Little is known about the role of matrix metalloproteinases (MMPs) and their inhibitors (TIMPs) in the vitreous and retinal environments. This study aimed to assess the vitreous levels of members of the MMP and TIMP families in patients who were scheduled to undergo pars plana vitrectomy (PPV). Prospectively, all patients scheduled for PPV and who met the inclusion criteria were invited. The included retinal conditions were advanced proliferative diabetic retinopathy (PDR), rhegmatogenous retinal detachment (RRD), vitreomacular interface diseases, endophthalmitis, and dropped crystalline lenses. Undiluted vitreous samples were obtained during the early stage of PPV. The levels of TIMP1, TIMP2, MMP2, and TIMP2/MMP2 ratio were measured using enzyme-linked immunosorbent assay (ELISA). A total of 181 eyes were included in this study. The levels of TIMP2 and the TIMP2/MMP2 ratio were significantly higher in the advanced PDR group than in the other groups. Significantly, TIMP2 and TIMP2/MMP2 levels were highest in the endophthalmitis group, whereas MMP2 levels were highest in the dropped crystalline lenses group. The presence of diabetes mellitus and of preoperative glaucoma were significantly associated with higher TIMP1 levels. In RRD cases alone, all biomarkers were significantly elevated with higher PVR grades. Furthermore, TIMP1 and MMP2 correlated with macular detachment. A relationship between the vitreous levels of MMPs and TIMPs and the pathogenesis of vitreoretinal pathology may exist. Further studies and trials are recommended to explore the potential use of MMPs and TIMPs in the diagnosis, prognosis, and treatment of eye diseases.

## 1. Introduction

Extracellular matrix processing is mediated by matrix metalloproteinases (MMPs), a large family of zinc-dependent endopeptidases with variable substrate spectra, which have been described in human vitreous and aqueous [1,2,3]. The activity and levels of these enzymes are regulated by specific endogenous inhibitors, the tissue inhibitors of metalloproteinases (TIMPs). Imbalance of the expression of MMPs and TIMPs has been implicated in many ocular disease processes accompanied by abnormal matrix production. Examples include diabetic retinopathy (DR), proliferative vitreoretinopathy (PVR), cataract, retinoblastoma, and glaucoma [4,5,6,7]. The vitreous is composed of a specialized extracellular matrix with gel-like consistency. The water content is very high (98% of the wet weight), and hyaluronan is the predominant glycosaminoglycan. Collagen types II, V, and IX are present and provide structural support to the gel [1].

The extracellular matrix (ECM) is an essential component of ocular tissue, contributing to the structure of basement membranes as well as specialized matrices such as the vitreous gel and the interphotoreceptor matrix. The homeostasis of the ECM is governed by a finely regulated equilibrium between its synthesis and degradation [8,9,10]. MMPs constitute a family of zinc-dependent endopeptidases enzymes that mediate the proteolytic degradation of structural ECM components, including collagens and proteoglycans. The activity of MMPs is tightly controlled by TIMPs, which bind to MMPs and inhibit their enzymatic function. Collectively, MMPs and TIMPs orchestrate the dynamic remodeling of the extracellular matrix in the eye [8,11,12]. MMPs are secreted as inactive proenzymes and undergo proteolytic activation by other MMPs or proteases, enabling them to interact with and degrade a wide array of ECM and cell surface proteins [8,12].

Within the ocular environment, MMPs are produced by several cell types, including fibroblasts, endothelial cells, and retinal pigment epithelial (RPE) cells, as well as infiltrating inflammatory cells such as macrophages, neutrophils, and lymphocytes. Their expression is regulated by numerous signaling molecules, including cytokines, growth factors, and hormones relevant to both physiological and pathological processes in the eye [13,14]. TIMP proteins, of which four isoforms (TIMP1 to TIMP4) have been identified, function as endogenous regulators that bind to active MMPs and inhibit their enzymatic activity [15].

Proliferative DR (PDR) is a common complication of diabetes mellitus (DM) characterized by preretinal neovascularization and development of epiretinal fibrovascular tissue [16]. Early features of DR include retinal capillary bed loss, which leads to damage to the inner blood–retinal barrier [16]. This process is followed by neovascularization, involving the production of angiogenic factors, as well as synthesis of the extracellular matrix (ECM) necessary for anchoring the migrating endothelium and other cells such as the retinal pigment epithelium (RPE), glial cells, and fibroblasts [16,17]. Little is known about the role of MMPs and TIMPs in the normal vitreous and in disease states involving the retina and vitreous, and minimal quantitative studies of vitreous levels of MMPs and TIMPs have been conducted.

This study aimed to investigate the levels of certain MMPs and TIMPs in the vitreous of patients underwent pars plana vitrectomy (PPV) for various ocular vitreoretinal disorders. Furthermore, the study assessed the factors that might affect the levels of these biomarkers.

## 2. Results

### 2.1. General Demographic and Clinical Characteristics with Associated Visual Outcomes

In this study, vitreous samples from 181 eyes of 177 patients were included. Of these, 115 (63.5%) samples were from men, and 98 eyes were right eyes (54.1%). The mean age of the patients was 53.3 years. General demographic characteristics are summarized in Table 1. When analyzing ocular parameters, the mean keratometry was 43.16 diopters, with no significant difference between the two groups (case and control groups). However, the control group had significantly longer axial lengths, with a mean of 24.98 mm compared to a mean of 23.61 mm in the case group (*p* = 0.02).

Comorbidities were also assessed, revealing significantly higher rates of DM, HTN and kidney diseases in the case group. Among patients with DM, the mean disease duration was 15.3 years. Of these, 41 (38.3%) were treated with oral hypoglycemic agents alone, while 66 (61.7%) received oral agents and/or insulin. The mean HBA1c percentage was significantly higher in the case group (7.69%) than in the control group (5.89%) (*p* = 0.0001).

The average time from presentation to surgery was 26.5 weeks in the case group, compared to 5.6 weeks in the control group (*p* = 0.0001). The primary indications for PPV were advanced PDR in 80 eyes, RRD in 65 eyes, vitreomacular interface disorders in 17 eyes, endophthalmitis in 5 eyes, and dropped IOL or crystalline lens in 14 eyes. Silicone oil was utilized as a tamponading agent in 95 eyes, gas in 49 eyes, and air in 37 eyes. The silicone oil was utilized more commonly in the control group, while the gas was utilized more frequently in the case group.

Preoperative glaucoma was documented in 26 eyes, 11 of which were in the case group. Postoperative use of anti-glaucoma medications was noted in 77 eyes, with the majority (49 eyes) in the control group (*p* = 0.047).

In assessing visual outcomes using LogMAR charts, both groups had a similar average preoperative BCVA of 1.502, with no significant intergroup difference. One month after surgery, the case group showed significantly worse BCVA (1.035 LogMAR units versus 0.840 LogMAR units in the control group) (*p* = 0.023). This improvement was more pronounced at three months postoperatively (0.006). Further details of the visual outcomes are summarized in Table 1.

Biochemical analysis and comparison of vitreous samples for TIMP1, TIMP2, MMP2, and the TIMP2/MMP2 ratio were performed between the case group and the control group. The mean concentrations and statistical comparisons are summarized in Table 2. There was no statistically significant difference in TIMP1 levels between the two groups. In contrast, TIMP2 levels were significantly elevated in the case group compared to the controls (29,310.8 pg/mg vs. 27,953.5 pg/mg; *p* = 0.021). Although MMP2 levels were slightly lower in the case group (15.4 ng/mL) compared to the control group (15.8 ng/mL), the difference was not statistically significant (*p* = 0.46). Importantly, the TIMP2/MMP2 ratio was significantly higher in the case group (1.93) than in the control group (1.90) (*p* = 0.043).

### 2.2. Comparative Analysis Among Retinal Disease Subgroups

A total of 181 eyes were stratified into the aforementioned five diagnostic and pathological subgroups of retinal disease and compared with demographic, systemic, surgical, and biochemical parameters (Table 3). There was a statistically significant difference in patient age across subgroups (*p* = 0.001), with the dropped IOL/lens group being the oldest (mean age, 65.2 years) and the endophthalmitis group being the youngest (mean age, 33.4 years). Sex distribution did not differ significantly. The prevalence of systemic comorbidities showed significant intergroup variation. DM, HTN, and chronic kidney diseases were most prevalent in the advanced PDR group. The duration of DM was longest in the advanced PDR group (17.4 years, *p* < 0.001).

Cataract surgery associated with the primary PPV was most frequently observed in the advanced PDR group (52.5%). The type of vitreous tamponade varied significantly among the groups (*p* = 0.001): silicone oil was most commonly used in the RRD (87.7%) group, gas tamponade was predominant in the VMT (82.4%) group, and air was frequently used in the dropped IOL/lens group (78.6%). Preoperative glaucoma was documented in 50.0% of dropped IOL/lens cases and 20.0% of endophthalmitis (the higher two groups), with a statistically significant difference among groups (*p* = 0.001). Postoperative anti-glaucoma medications showed a highly significant intergroup difference (*p* = 0.01), being most frequent in RRD (56.9%), followed by dropped IOL/lens (50.0%).

Significant differences were observed in vitreous biomarker levels among the retinal disease subgroups. TIMP1 levels were highest in the endophthalmitis group (213,717.8 pg/mL) but did not reach statistical significance across groups (*p* = 0.12). TIMP2 concentrations differed significantly (*p* = 0.003), with the endophthalmitis group exhibiting the highest levels (64,800 pg/mg). MMP2 levels were also significantly different (*p* = 0.03), with the dropped IOL/lens group showing the highest concentration (19.2 ng/mL) and VMT the lowest (9.9 ng/mL). The TIMP2/MMP2 ratio varied significantly among groups (*p* = 0.004), being highest in the endophthalmitis group (6.49) and lowest in the dropped IOL/lens cases (1.46). This suggests a disease-specific imbalance between proteolytic activity and its inhibition.

### 2.3. Factors Affecting the Level of Each Biomarker Separately in the Whole Sample

The factors influencing the levels of TIMP1, TIMP2, MMP2, and TIMP2/MMP2 ratio in the study were evaluated (Table 4). Regarding TIMP1, age, gender, and ocular parameters had no effect on the levels of TIMP1. The study revealed that the patients with DM had higher levels of TIMP1 (119,801.2 pg/mL, *p* = 0.036). However, the type of DM treatment, the duration of DM, and the level of HBA1c had no effect. Moreover, the type of diagnostic pathology, duration from presentation to surgery, associated cataract surgery with primary PPV, and the type of vitreous tamponade had no influence on the levels of TIMP1. The presence of preoperative glaucoma significantly increased the levels of TIMP1 (176,623.6 pg/mL, *p* = 0.018). On multiple regression analysis, it was revealed that the presence of DM and preoperative glaucoma were independent risk factors affecting TIMP1 levels.

Regarding the TIMP2 levels, age, gender, and ocular parameters had no effect on the levels of TIMP2. Patients who had chronic kidney disease had higher levels of TIMP2 (33,453.5 pg/mL, *p* = 0.043). Although the presence of DM and HTN had no effect, the type of DM treatment significantly affected the levels of TIMP2, in which the levels of TIMP2 for patients using oral hypoglycemic agents alone was 22,961.4 pg/mL, and for those using oral hypoglycemic agents and/or insulin, it was 30,459.1 pg/mL (*p* = 0.007). Furthermore, the longer the duration of DM, the higher the levels of TIMP2 (for an increase of one year of DM duration, the level of TIMP2 was elevated by 534.6 pg/mL, *p* = 0.004). Diagnostic retinal pathology was found to significantly influence TIMP2 levels. Patients with endophthalmitis and advanced PDR had the highest levels of TIMP2 (64,800.0 pg/mL and 29,310.8 pg/mL, respectively, *p* = 0.003). Moreover, the study revealed that the duration of presentation to surgery, associated cataract surgery with primary PPV, presence of preoperative of postoperative glaucoma, type of vitreous tamponade and the level of HBA1c had no correlation with TIMP2 levels.

The MMP2 levels were not associated with age, gender, or ocular parameters. Patients who had chronic kidney disease had higher levels of MMP2 (17.3 ng/mL, *p* = 0.039). Furthermore, the presence of DM, the type of treatment of DM, the duration of DM, and the level of HBA1c had no significant effect on MMP2 levels. Preoperative diagnostic pathology significantly influenced the levels of MMP2, and it was highest in patients with dropped IOL/crystalline lens followed by patients with endophthalmitis (19.2 ng/mL and 17.0 ng/mL, respectively, *p* = 0.03). Preoperative glaucoma was found to be associated with MMP2 levels. Duration of presentation for surgery, associated cataract surgery with primary PPV, type of vitreous tamponade, and the presence of glaucoma postoperatively had no significant effect on MMP2 levels. Multiple regression analysis revealed that the presence of preoperative glaucoma was the independent risk factor affecting MMP2 levels.

Regarding the TIMP2/MMP2 ratio, the age, gender, ocular parameters and presence of co-morbidities had no influence. The study revealed that the type of DM treatment significantly affected the ratio in which patients who used oral hypoglycemic agents alone, the ratio was 1.52, and the ratio of those who used oral hypoglycemic agents and/or insulin was 2.01 (*p* = 0.001). Furthermore, the duration of DM had a statistically significant effect on the TIMP2/MMP2 ratio (for an increase of one year of DM duration, the level of the TIMP2/MMP2 ratio was elevated by 0.04, *p* = 0.0001). Furthermore, preoperative diagnostic retinal pathology significantly influenced the TIMP2/MMP2 ratio, in which the highest ratio was observed in patients with endophthalmitis (6.49) and the lowest ratio was observed in patients with dropped IOL/crystalline lens (1.46, *p* = 0.004). The duration of presentation for surgery, associated cataract surgery with primary PPV, presence of glaucoma, and type of tamponade had no effect. Multiple regression analysis revealed that the longer the duration of DM and the endophthalmitis diagnosis was associated with TIMP2/MMP2 ratio.

### 2.4. Factors Affecting the Levels of Biomarkers in RRD Patients

A sub-analysis was performed on patients with RRD (*n* = 65) (Table 5). Age and gender showed no significant association with the levels of any of the biomarkers. Similarly, comorbidities such as DM, HTN, and chronic kidney disease demonstrated no significant correlations. Moreover, no significant associations were observed between biomarker levels and axial length, keratometry, and the duration of RRD presentation. However, macular status was significantly associated with TIMP1 and MMP2 levels. Both biomarkers were elevated in the detached macula group, with mean TIMP1 levels of 94,272.4 pg/mL compared to 69,577.4 pg/mL in the attached macula group (*p* = 0.02), and mean MMP2 levels of 18.7 ng/mL in the detached macula group versus 12.4 ng/mL in the attached macula group (*p* = 0.01).

The location of the retinal tear had no significant influence on biomarker levels. Despite this, a trend toward higher TIMP1 levels was noted in eyes with inferior retinal tears (mean = 128,037.9 pg/mL) than in those with superior retinal tears (mean = 61,062.1 pg/mL), although this was not statistically significant (*p* = 0.08). A strong association was found between the number of quadrants involved and TIMP1 levels. Eyes with the involvement of more than two quadrants had significantly higher TIMP1 levels (mean = 115,939.1 pg/mL) than those with one or two quadrants involved (mean = 61,176.6 pg/mL; *p* = 0.0001). Similarly, both TIMP2 and MMP2 levels were significantly elevated in cases involving more than two quadrants (*p* = 0.001 for both).

Furthermore, patients with advanced PVR (PVR grades C or D) exhibited significantly higher levels of TIMP1, TIMP2, and MMP2 than those with early-stage PVR (grades A or B). The presence of any grade of PVR was also associated with significantly higher biomarker levels compared to eyes without PVR. No significant correlations were found between biomarker levels and the presence of associated vitreous hemorrhage, preoperative glaucoma, or the postoperative use of antiglaucoma medications. Multiple regression analysis revealed that a higher grade of PVR was independently associated with higher levels of TIMP2. This is similar for MMP2 levels. Furthermore, higher grades of PVR and larger numbers of quadrants involved were independent factors for TIMP1 levels.

### 2.5. Factors Affecting the Levels of Biomarkers in Advanced PDR Patients

Further sub-analysis was performed on patients with advanced PDR alone (*n* = 80). A significant association was detected between insulin use and TIMP2 levels (*p* = 0.026). Furthermore, the presence of preoperative glaucoma was associated with higher levels of TIMP2 and MMP2. Moreover, the presence of intraoperative diabetic ERM correlated with higher levels of TIMP1 and TIMP2. Additionally, the longer the duration of DM, the higher the levels of TIMP2. No correlations were detected between biomarkers levels and the severity of advanced PDR stages (such as the degree of TRD, the presence of tears, or extensive VH). Moreover, the number of intravitreal anti-VEGF injections and the duration from the last intravitreal anti-VEGF injection were not associated with biomarker levels.

## 3. Discussion

This study was conducted to investigate the levels of certain MMPs and TIMPs in various ocular conditions and to assess the factors that may affect their levels. To the best of our knowledge, this is the first study in Jordanian populations and the first study to investigate five various ocular retinal pathologies (advanced PDR, RRD, vitreomacular interface diseases, endophthalmitis, and dropped lens). This study revealed that TIMP2 and the TIMP2/MMP2 ratio were significantly elevated in advanced PDR cases (case group). Moreover, endophthalmitis cases exhibited higher levels of TIMP2, whereas vitreomacular interface disorders showed lower levels of TIMP2. In addition, the TIMP2 levels and TIMP2/MMP2 ratio increased in patients with a longer DM duration and those who received insulin treatment. Furthermore, TIMP1 and MMP2 levels were significantly elevated in patients with preoperative glaucoma. Interestingly, MMP2 levels were significantly elevated in patients with chronic kidney diseases and increased significantly in patients with a dropped crystalline lens. In RRD cases, the three biomarkers were significantly elevated in cases of more extensive detachment and higher PVR grades. Furthermore, TIMP1 and MMP2 correlated with the detachment of the macula. In advanced PDR cases, the presence of diabetic ERM was associated with higher TIMP1 and TIMP2 levels.

A disruption in the balance between MMPs and TIMPs can significantly alter ECM turnover, potentially contributing to pathological changes in ocular tissues, such as those observed in retinal detachment, cataract, retinoblastoma, glaucoma, age-related macular degeneration, and PVR [12,18,19,20,21].

Regarding the role of TIMPs and MMPs in diabetic retinopathy, Ünal et al. investigated the levels of MMP9 and MMP14 in the PDR group and compared the levels to the non-PDR group [22]. They found that the mean MMP9 and MMP14 levels in the PDR group were significantly higher than the non-PDR group (*p* < 0.01). Also, they observed that MMP9 and MMP14 levels increased with a longer duration of DM [22]. Another study by Matsuo et al. was conducted to measure the vitreous and subretinal levels of TIMP1 and TIMP2 [8]. They found that TIMP1 levels in vitreous fluid in PDR and PVR patients were higher than those in vitreous fluid with other diseases [8]. Another study by Jin et al. was correlated with these results in which MMP9 was significantly higher in DR samples [23]. These findings were consistent with findings of the study by El-Asrar et al. in which MMP1, MMP7, and MMP9 levels were higher in PDR patients than in non-diabetic patients [24]. Kowluru et al. revealed that the elevated levels of MMP9 in PDR cases increased oxidative stress in retinal capillary cells via mitochondria and consequently accelerated apoptosis in retinal capillary cells [25]. Elevated levels of MMP2 and MMP9 activity were demonstrated in glucose-induced rhesus macaque choroid–retinal endothelial cells and bovine retinal endothelial cells, and these effects seem to be mediated by the activation of the Akt and ERK pathways [26]. Furthermore, the levels of TIMP1, TIMP4, MMP1, MMP-9, and MMP-14 were revealed by all El-Asrar studies to be significantly correlated with VEGF levels, the principal angiogenic factor in PDR [24,27]. The angiogenic switch partly depends on the breakdown of basement membranes and ECM components by MMPs. These enzymes not only eliminate structural barriers to new blood vessel formation but also release VEGF from its stores within the ECM. This release boosts the availability of VEGF, thereby initiating the VEGF-mediated angiogenic switch. The action of MMP2 takes part in the breakdown of the blood–retinal barrier by degrading proteins, resulting in increased vascular permeability and blood–retinal barrier disruption, and this breakdown is associated with the occurrence of diabetic macular edema, which is a critical hallmark of DR [13]. A tissue membranes study was conducted by Salzmann et al. and concluded that unlike normal retina, which constitutively expressed MMP1 and TIMP2, a large proportion of PDR membranes stained for MMP1, MMP2, MMP3, MMP9, TIMP1, TIMP2, and TIMP3. There were no differences in the expression of these molecules when compared with PVR membranes. There was a significant increase in TIMP2 expression by PDR membranes when compared with PVR membranes [16].

Elevated concentrations of TIMP1 observed in the subretinal fluid of patients with RRD may reflect an upregulation of TIMP1 expression by RPE cells, potentially as a protective mechanism aimed at preserving the integrity of the interphotoreceptor matrix and, consequently, photoreceptor viability [8]. In chronic cases of RRD, the subretinal fluid is frequently characterized by elevated total protein levels, a consequence of active fluid resorption by RPE cells. Therefore, the increased TIMP1 levels detected in such cases may alternatively be attributed to passive protein concentration rather than active synthesis [8]. A study was conducted by Kon et al. on 140 cases of RRD patients [4]. They found that MMP-2 was observed in all of the vitreous samples obtained, while MMP was observed in 64 samples. The levels of MMPs detected were not significantly associated with the presence of preoperative PVR; however, they were significantly associated with the development of postoperative PVR [4]. Symeonidis et al. investigated the levels of MMP2, MMP9, MMP1, MMP3, MMP8, and TIMP1 in the vitreous and subretinal fluid of RRD patients and control subjects [28]. They concluded that MMPs and TIMP1 were differentially present in subretinal fluid and the vitreous. PVR grade correlated significantly with the levels of MMP2 in the subretinal fluid, while MMP1, MMP2, MMP3, MMP8, MMP9 and TIMP1 levels correlated with the PVR grade in the vitreous [28]. In the in vivo PVR membranes, it is likely that RPE cells produce MMPs, which then may play a crucial role in the contraction of the membrane. PVR membranes develop mainly in the inferior retina where the retinal RPE cells settle [4]. In this study, the three biomarkers were elevated significantly in cases of more quadrants of involvement and with higher PVR grades. Furthermore, TIMP1 and MMP2 correlated with the detachment of the macula. The biomarkers showed a trend toward inferior tears.

Interestingly, this study was the first work to correlate the vitreous (not the aqueous) levels of MMPs and TIMPs with glaucoma. We found that the levels of TIMP1 and MMP2 were higher in patients with glaucoma. Schlötzer-Schrehardt et al. measured the activity and levels of MMP1, MMP2, MMP3, MMP7, MMP9, MMP12, TIMP1, and TIMP2 in the aqueous humor of patients with pseudoexfoliative glaucoma, primary open-angle glaucoma, and cataract [3]. They found that MMP2, MMP3, TIMP1, and TIMP2 were detected at significantly higher concentrations in aqueous samples from eyes with pseudoexfoliation with and without glaucoma compared with cataractous eyes. MMP2, MMP3, and TIMP1 were also detected in higher, but not significantly different, amounts in aqueous samples of primary open-angle glaucoma eyes [3]. According to Markiewicz et al., it was revealed that patients with primary open-angle glaucoma had higher levels of MMP1, MMP2, MMP3, MMP9, and MMP12 in the aqueous humor compared to the controls without any type of glaucoma [29]. MMP2, known as gelatinase-A, may play a similar role as MMP9, in which it has been demonstrated that the MMP9 null mice had aberrant collagen composition of the trabecular meshwork, as well as lowered aqueous humor turnover and ocular hypertension, indicating that MMP9 may be an important remodeler of trabecular meshwork, mitigating the course of glaucoma [30].

Practically, MMP pharmacological inhibitors may be promising agents that may have an influence on many ocular diseases. Such examples of medications or agents that target the MMPs include doxycycline, which broadly inhibits many MMPs [31,32]. One of the antihelminthics, niclosamide, is a salicylanilide that is able to inhibit uveal melanoma [33]. Niclosamide treatment limited the invasion and migration of uveal melanoma cells, and decreased MMPs protein expression [34]. Theoretically, pharmacological inhibitors of MMPs may play a role in the prevention of the development of DR and protect against vascular permeability and inflammation [35]. Also, the use of synthetic MMP inhibitors may prevent the induction of PVR, which complicates any retinal surgery [36]. However, there is no clinical trial on the utilization of MMP inhibitors.

There are few studies in literature investigating the factors affecting MMPs and TIMPs in the vitreous. Furthermore, many of these studies used bioequivalent living vitreous, while this study utilized human vitreous. The sample size in this study is relatively large. Moreover, the study comprised various retinal diseases in the analyses along with multiple patients and medical and ocular co-factors. However, no study is without limitations. As the variability of ocular conditions is considered a strengthening point, it is actually considered a limitation point. In addition, only TIMP1, TIMP2, and MMP2 were analyzed, many other MMPs and TIMPs may have a role in the ocular diseases. Third, the study had no classification for glaucoma and its types.

In conclusion, the findings of this study may indicate a relationship between the MMP and the TIMP families and the development of certain vitreoretinal ocular conditions. Both MMPs and TIMPs may play crucial roles in the pathogenesis and progression of PDR, PVR formation, vitreomacular interface disease, endophthalmitis, and glaucoma. Furthermore, their expression and activity may be influenced by other co-factors. Furthermore, the ratio of MMP/TIMP levels may be as important as the individual levels of these biomarkers. We recommend further studies and trials to assess the role and potential use of MMPs and TIMPs in the diagnosis, prognosis, and treatment of eye diseases. In addition, further studies are needed to identify novel, therapeutic options modulating MMP and TIMP expression and activity, and these may slow the prevalence, development, and progression of eye diseases.

## 4. Materials and Methods

### 4.1. Patients and Data

After obtaining the required ethical approval, vitreous samples were collected from patients who underwent PPV for any indication between October 2021 and June 2024. All patients who presented with the complaint of visual impairment and were scheduled for PPV with vitreoretinal pathology in the ocular examination were included in this study. The study was implemented at the Ophthalmology department at King Abdullah University Hospital (KAUH) and at the laboratories of Jordan University of Science and Technology. Written informed consent was obtained from all participants, and the study was conducted according to the tenets of the Declaration of Helsinki. Before the study, the participants were given detailed information about the purpose of the study and the procedures to be performed. Following a comprehensive review of the medical record, patients’ demographics and medical history were documented. The use of anti-glaucoma agents preoperatively and postoperatively was addressed. Furthermore, the previous ocular surgery, details of intravitreal anti-vascular endothelial growth factor (VEGF) injection, and panretinal photocoagulation laser sessions were allocated. Moreover, operative and postoperative information, including details of the vitreoretinal pathology, grading of the retinal findings, associated operative procedures, the type of utilized tamponade that was obtained, and the postoperative visual acuity.

The primary outcome of the study was to measure and assess the factors influencing the vitreous levels of TIMP1, TIMP2, MMP2, and the TIMP2/MMP2 ratio. Secondary outcomes included the variations between vitreoretinal pathology, and their anatomical association with the aforementioned biomarkers.

All patients who presented with vitreoretinal pathology that necessitated PPV were enrolled in the study. Eyes with the following conditions were excluded: a previously vitrectomized patient; penetrating eye injury; recent vitreous hemorrhage (VH) within two months; topical or systemic steroid treatment, where the procedure of obtaining vitreous sample may result in serious complications; and prior chemotherapy administration.

The pathologies requiring PPV surgery were categorized within the following groups. The first was the advanced DR group, which comprised all diabetic patients in the proliferative diabetic retinopathy (PDR) stages and were unresponsive to medical treatment. These advanced PDR stages included traction retinal detachment (TRD) from fibrovascular membranes (FVM) proliferation, combined traction/rhegmatogenous retinal detachment, and/or non-clearing VH. DR was graded according to the modified Airlie house classifications [37]. The second group of participants was rhegmatogenous retinal detachment (RRD) with or without PVR. The third group comprised patients with vitreomacular interface diseases with included macular hole (MH), vitreomacular traction (VMT), and non-diabetic epiretinal membranes (ERM). The fourth group was those who were operated on as a result of previous complicated cataract surgery (either a drop of the crystalline lens or its parts or a drop of intraocular lens implants (IOLs)). The last group was endophthalmitis cases. Those pathologies were also categorized into a case group, which comprised the advanced PDR cases, and the control group consisted of the other groups of pathology. Control groups may include diabetic patients; however, their fundi did not exhibit DR.

The investigated past medical history encompassed details of DM status (duration of DM, treatment of DM, and the level of last HbA1c), and the presence of hypertension, and chronic kidney diseases. Intravitreal anti-VEGF injections were utilized in most patients in the case group and in a minority of the control group (cases of ERM or VMT) preoperatively. The number, type, and duration of the last injection were included in the analysis. Either aflibercept or ranibizumab was utilized in this study. In addition, the effect of panretinal photocoagulation laser sessions was assessed. Hba1c and homocysteine levels were measured for all participants.

A sub-analysis was run for RRD cases alone to correlate RRD characteristics with the vitreous levels of the biomarkers. The assessed characteristics of RRD were macular status (either the macula was attached or not), the location of the tear (superiorly or inferiorly), the number of quadrants of involvement, the grade of PVR, and the duration of symptoms of RRD. PVR was graded according to the Retina Society Terminology Committee (1983) classification [38].

Another sub-analysis was performed for advanced PDR cases alone. The investigated features of the advanced PDR cases were the presence of VH, the presence of TRD, the occurrence of tears (complex retinal detachment), and the development of diabetic ERM or MH.

### 4.2. Perioperative Settings and Sample Collection

Snellen visual acuity was established for each participant on the day of admission, one month and three months postoperatively, and the last follow-up visit. The visual acuity was then converted to the LogMAR best-corrected visual acuity (BCVA). Anterior and posterior segment examination was performed through slit-lamp biomicroscopy with the required non-contact hand-held lenses. Intraocular pressure (IOP) measurement was performed with applanation tonometry.

As per our previous experiments [17,39], a single vitreoretinal consultant was responsible for PPV and its related procedures and for vitreous sample collection. Under general or local anesthesia, the conjunctival sac was washed with povidone iodine 5%. Three trans-sclerotomies were created through 23-gauge trocars (Combined Wide-Field Elite Pack, Bausch, and Lomb) 3.5–4 mm posterior to the surgical limbus at the superior-temporal, inferior–temporal, and superior–nasal quadrants. Then, an undiluted 1–2 mL vitreous sample was collected from the mid-vitreous using the vitreous cutter (a cut rate of 5000 cuts per minute) without an infusion to prevent dilution of the sample. The required vitreous samples were considered as surgical byproducts in which the surgical procedure performed will not be altered or modified, preserving the safety of the patient with optimum surgical efficacy. Following this step, the PPV surgery was completed by inserting the infusion cannula at the inferior–temporal port, and subsequent retinal procedures according to the indications and operative findings were performed. Silicone oil, sulfur hexafluoride (SF6) gas, and air were utilized as a tamponade.

Vitreous samples were then transferred into 2 mL sterile tubes (Eppendorf, Freemont, CA, USA). They were kept at −80 °C and were defrosted only in small quantities for each analysis. The samples were labeled by the names of the participants and the hospital identification numbers and were given assigned serial numbers.

### 4.3. Sample Processing and Biomarker Measurement

TIMP1, TIMP2, TIMP3, MMP1, MMP2, MMP3, and MMP9 were investigated, and the concentrations were determined using an enzyme-linked immunosorbent assay (ELISA) (R&D Systems, Minneapolis, MN, USA), executed in accordance with the manufacturer’s instructions. The solid-phase sandwich ELISA technique was adopted. Quality control samples and random analysis of the vitreous samples were performed. From initial investigation test analysis, only TIMP1 (DY970), TIMP2 (DY971), and MMP2 (DY902) were detected in the vitreous samples. Depending upon the detection range for each ELISA kit, the supernatant vitreous (after being centrifuged at 20,000× *g* for 15 min at 4 °C) obtained was used either directly or diluted with calibrator diluents supplied to the ELISA kit. The minimum detection range of the employed ELISA was TIMP1: 31.2 pg/mL, TMP2: 31.2 pg/mL, and MMP2: 0.6 ng/mL. The appropriate sample dilution was as follows: TIMP1 and TIMP2: 1:200, and MMP2: 1:1.

After incubation, washing, and coating the ELISA plates (for all included biomarkers) with the capture antibody, an amount of 100 μL of the aforementioned diluted vitreous fluid was added to each well of the ELISA plate. The assay was performed in duplicate for each standard and vitreous sample. Antibodies against TIMP1, TIMP2, and MMP2 were added to each well of the ELISA plate. After the addition of the substrate mix solution, the reaction was halted by adding the stop solution after the development of color. Optical Density was read at 450 nm with the use of a microplate reader with wavelength correction of 540 nm to 570 nm using an absorption spectrophotometer (Bio-Tek Instruments, Winooski, VT, USA). A standard curve was generated out of measurements made with the standard solution and was used to determine the concentrations in each sample. The standardized ratio between TIMP2 and MMP2 was calculated and measured for associated factors.

### 4.4. Statistical Analysis

Raw data were analyzed using the IBM statistical package for the social sciences (SPSS) v.26 (Armonk, New York, NY, USA). Data were expressed as frequency (percentage) or mean ± standard error of the mean (SEM). To verify normality, the Kolmogorov–Smirnov test was performed. It was found that the biomarkers levels were abnormally distributed. Accordingly, for continuous variables, Kruskal–Wallis’s test was employed to detect statistical significance, and the mean rank, along with the mean and SEM, was utilized for expression in our tables. For categorical variables, the statistical significance between the study groups was determined using the Chi-square test. Simple linear regression analysis was employed to estimate the effect size between two continuous variables (expressed as the B coefficient). Multiple logistic regression analyses were implemented to evaluate the predictive potential of TIMP1, TIMP2, and MMP2. *p*-values ≤ 0.05 were considered statistically significant.

## Figures and Tables

**Table 1:** Demographical details of study population (*n* = 181) and comparison between case and control groups.

Variables	Number (Percentage) or Mean ± SEM (Mean Rank)			
	Overall (*n* = 181)	Case Group (*n* = 80)	Control Group (*n* = 101)	*p*-Value
**Age**	53.3 ± 1.1	52.8 ± 1.2 (84.5)	53.8 ± 1.7 (96.1)	0.66
**Gender**				
Male	115 (63.5)	46 (57.5)	69 (68.3)	0.08
Female	66 (36.5)	34 (42.5)	32 (31.7)	
**Eye laterality**				
Right eye (OD)	98 (54.1)	42 (52.5)	56 (55.4)	0.40
Left eye (OS)	83 (45.9)	38 (47.5)	45 (44.6)	
**Ocular parameters**				
Axial length (mm)	24.34 ± 0.2	23.61 ± 0.4 (38.3)	24.98 ± 0.3 (67.8)	0.02
Km (Diopter)	43.16 ± 0.3	43.44 ± 0.2 (59.5)	42.90 ± 0.1 (50.0)	0.39
**Co-morbidities**				
DM	107 (59.6)	78 (97.5)	29 (28.7)	0.0001
Hypertension	102 (56.4)	60 (75.0)	42 (41.6)	0.001
End-stage renal disease	17 (9.4)	14 (17.5)	3 (3.0)	0.001
**Treatment of DM**				
Oral hypoglycemic agents	41 (38.3)	22 (28.2)	19 (65.5)	0.001
Oral hypoglycemic agents and/or insulin	66 (61.7)	56 (71.8)	10 (34.5)	
**Duration of DM (years)**	15.3 ± 0.7	17.4 ± 0.8 (54.7)	8.9 ± 1.0 (27.0)	0.0001
**Preoperative diagnosis**				
Advanced PDR (FVM and VH)	80 (44.2)	80 (100.0)	0 (0.0)	
Rhegmatogenous Retinal Detachment	65 (35.9)	0 (0.0)	65 (64.4)	
Vitreomacular interface diseases (ERM/MH/VMT)	17 (9.4)	0 (0.0)	17 (16.8)	0.0001
Endophthalmitis	5 (2.8)	0 (0.0)	5 (5.0)	
Dropped IOL or crystalline lens	14 (7.7)	0 (0.0)	14 (13.9)	
**Associated cataract surgery with the primary PPV**	65 (35.9)	42 (52.5)	23 (22.8)	0.0001
**Duration from presentation for surgery (weeks)**	15.5 ± 1.8	26.5 ± 3.1 (112.8)	5.6 ± 1.2 (53.2)	0.0001
**Type of vitreous tamponade**				
Silicone oil	95 (52.5)	32 (40.0)	63 (62.4)	
Gas	49 (27.1)	28 (35.0)	21 (20.8)	0.011
Air	37 (20.4)	20 (25.0)	17 (16.8)	
**Preoperative glaucoma**	26 (14.4)	11 (13.8)	15 (14.9)	0.50
**Postoperative AG use**	77 (42.5)	28 (35.0)	49 (48.5)	0.047
**Visual outcomes (Log MAR)**				
Preoperative BCVA	1.502 ± 0.03	1.582 ± 0.04 (97.6)	1.437 ± 0.06 (84.8)	0.21
BCVA after 1 month	0.926 ± 0.01	1.035 ± 0.03 (99.1)	0.840 ± 0.02 (82.0)	0.023
BCVA after 3 months	0.947 ± 0.02	1.108 ± 0.03 (85.4)	0.831 ± 0.01 (68.6)	0.006
BCVA at the last follow-up	0.911 ± 0.01	1.038 ± 0.05 (96.5)	0.809 ± 0.02 (83.9)	0.025
**HBA1c (%)**	7.69 ± 0.2	9.73 ± 0.3 (108.8)	5.89 ± 0.2 (46.3)	0.0001
**Homocystein (mcmol/L)**	22.0 ± 2.5	21.5 ± 4.2 (28.9)	22.6 ± 2.6 (34.2)	0.84

**Abbreviations:** DM: diabetes mellitus; PDR: proliferative diabetic retinopathy; VH: vitreous hemorrhage; ERM: epiretinal membrane; VMT: vitreomacular traction; MH: macular hole; AG: anti-glaucoma; PPV: pars plana vitrectomy; SEM: standard error of mean.

**Table 2:** Measured biomarkers in vitreous samples in both groups.

Measured Markers	Mean ± SEM (Mean-Rank)			
	Overall (*n* = 181)	Case Group (*n* = 80)	Control Group (*n* = 101)	*p*-Value
TIMP1 (pg/mL)	107,474.5 ± 1029.1	113,284.8 ± 1595.1 (89.7)	102,909.9 ± 1359.6 (86.7)	0.69
TIMP2 (pg/mg)	28,553.2 ± 739.7	29,310.8 ± 883.4 (100.3)	27,953.5 ± 745.1 (83.6)	0.021
MMP2 (ng/mL)	15.6 ± 0.7	15.4 ± 0.6 (94.3)	15.8 ± 0.5 (88.4)	0.46
TIMP2/MMP2 ratio	1.92 ± 0.1	1.93 ± 0.08 (99.8)	1.90 ± 0.07 (83.9)	0.043

**Abbreviations:** SEM: standard error of mean; TIMP: tissue inhibitor of metalloproteinase; MMP: matrix metalloproteinase.

**Table 3:** A comparative analysis between all groups of retinal diseases.

Variables	Number (Percentage) or Mean ± SEM (Mean Rank)					
	Advanced PDR (*n* = 80)	RRD (*n* = 65)	Vitreomacular Disease (*n* = 17)	Endophthalmitis (*n* = 5)	Dropped IOL/Lens (*n* = 14)	*p*-Value
**Age**	52.8 ± 1.2 (84.5)	51.4 ± 1.4 (85.5)	59.6 ± 0.9 (119.7)	33.4 ± 11.8 (47.1)	65.2 ± 2.7 (134.3)	0.001
**Gender**						
Male	46 (57.5)	47 (72.3)	11 (64.7)	1 (20.0)	10 (71.4)	0.09
Female	34 (42.5)	18 (27.7)	6 (35.3)	4 (80.0)	4 (28.6)	
**Co-morbidities**						
Diabetes mellitus	78 (97.5)	15 (23.1)	6 (35.3)	0 (0.0)	8 (57.1)	0.001
Hypertension	60 (75.0)	22 (33.8)	8 (47.1)	2 (40.0)	10 (71.4)	0.001
Chronic kidney disease	14 (17.5)	1 (1.5)	1 (5.9)	0 (0.0)	1 (7.1)	0.019
**Duration of diabetes mellitus (years)**	17.4 ± 0.5 (54.7)	6.6 ± 0.2 (18.5)	15.3 ± 0.6 (51.0)	-	10.6 ± 0.7 (31.9)	0.001
**Associated cataract surgery with the primary PPV**	42 (52.5)	10 (15.4)	7 (41.2)	2 (40.0)	4 (28.6)	0.001
**Duration from presentation for surgery (weeks)**	26.5 ± 3.1 (112.8)	2.8 ± 1.1 (38.1)	16.9 ± 1.5 (97.8)	0.6 ± 0.1 (29.0)	4.0 ± 1.4 (64.6)	0.001
**Type of vitreous tamponade**						
Silicone oil	32 (40.0)	57 (87.7)	1 (5.9)	3 (60.0)	2 (14.3)	
Gas	28 (35.0)	4 (6.2)	14 (82.4)	2 (40.0)	1 (7.1)	0.001
Air	20 (25.0)	4 (6.2)	2 (11.8)	0 (0.0)	11 (78.6)	
**Preoperative glaucoma**	11 (13.8)	4 (6.2)	3 (17.6)	1 (20.0)	7 (50.0)	0.001
**Postoperative AG use**	28 (35.0)	37 (56.9)	3 (17.6)	2 (40.0)	7 (50.0)	0.017
**HBA1c (%)**	9.7 ± 0.4 (108.8)	5.4 ± 0.2 (37.4)	6.7 ± 0.6 (60.7)	4.9 ± 0.1 (4.5)	7.1 ± 0.6 (43.3)	0.001
**Homocysteine (mcmol/L)**	21.5 ± 4.2 (28.9)	24.2 ± 2.4 (36.6)	24.6 ± 2.7 (37.8)	20.9 ± 5.6 (29.5)	14.9 ± 1.8 (23.6)	0.45
**Vitreous biomarkers**						
TIMP1 (pg/mL)	113,284.7 ± 5957.1 (89.7)	85,907.2 ± 2597.1 (81.9)	71,862.7 ± 1409.6 (76.4)	114,542.9 ± 3973.5 (87.8)	21,1751.8 ± 8758.2 (119.5)	0.12
TIMP2 (pg/mg)	29,310.8 ± 883.3 (100.3)	28,714.9 ± 375.1 (86.7)	15,182.4 ± 532.1 (50.4)	64,800.0 ± 8356 (127.6)	26,763.1 ± 739 (94.4)	0.003
MMP2 (ng/mL)	15.4 ± 0.7 (94.3)	16.5 ± 0.5 (91.2)	9.9 ± 1.1 (56.0)	17.0 ± 1.2 (90.0)	19.2 ± 1.1 (114.4)	0.03
TIMP2/MMP2 ratio	1.93 ± 0.07 (99.8)	1.72 ± 0.08 (83.9)	1.64 ± 0.08 (78.8)	6.49 ± 2.6 (156.0)	1.46 ± 0.1 (64.9)	0.004

**Abbreviations:** PDR: proliferative diabetic retinopathy; RRD: rhegmatogenous retinal detachment; AG: anti-glaucoma; PPV: pars plana vitrectomy; SEM: standard error of mean; TIMP: tissue inhibitor of metalloproteinase; MMP: matrix metalloproteinase.

**Table 4:** Factors affecting the level of each biomarker separately in the whole sample.

Variables	Mean ± SEM (Mean Rank) or B Regression Coefficient ± SEM							
	TIMP1 (pg/mL)	*p*-Value	TIMP2 (pg/mg)	*p*-Value	MMP2 (ng/mL)	*p*-Value	TIMP2/MMP2 Ratio	*p*-Value
**Age**	1236.7 ± 876	0.07	−74.3 ± 115	0.52	−0.23 ± 0.47	0.63	−0.01 ± 0.07	0.16
**Gender**								
Male	98,534.4 ± 1294 (87.3)	0.41	27,491.2 ± 280 (88.6)	0.43	15.3 ± 1.1 (88.7)	0.80	1.84 ± 0.07 (90.6)	0.89
Female	12,2981.1 ± 1216 (89.7)		30,402.5 ± 352 (95.2)		16.1 ± 0.9 (95.1)		2.06 ± 0.2 (91.6)	
**Ocular parameters**								
Axial length (mm)	−3349.9 ± 121	0.42	565.4 ± 706	0.42	0.05 ± 0.3	0.87	0.02 ± 0.01	0.30
Km (Diopter)	−558.2 ± 811	0.88	−217.7 ± 654	0.74	0.04 ± 0.3	0.88	−0.01 ± 0.02	0.39
**Co-morbidities**								
DM	119,801.2 ± 4373 (93.5)	0.036	27,586.2 ± 577 (94.5)	0.19	15.5 ± 0.7 (94.0)	0.19	1.82 ± 0.06 (90.9)	0.60
Hypertension	112,793.1 ± 4321 (90.9)	0.91	27,620.6 ± 117 (90.4)	0.72	15.0 ± 0.8 (90.0)	0.72	1.90 ± 0.09 (91.1)	0.63
Chronic kidney disease	149,788.9 ± 6275 (111.0)	0.057	33,453.5 ± 628 (115.5)	0.043	17.3 ± 1.4 (108.8)	0.039	1.96 ± 0.1 (109.7)	0.12
**Treatment of DM**								
Oral hypoglycemic agents	141,009.3 ± 6226 (56.7)	0.94	22,961.4 ± 189 (45.2)	0.007	15.2 ± 1.1 (56.7)	0.22	1.52 ± 0.07 (41.3)	0.001
Oral hypoglycemic agents and/or insulin	107,076.4 ± 6739 (50.0)		30,459.1 ± 100 (59.5)		15.6 ± 0.8 (49.9)		2.01 ± 0.09 (61.8)	
**Duration of DM (years)**	2237.7 ± 901	0.24	534.6 ± 179	0.004	0.064 ± 0.08	0.45	0.04 ± 0.008	0.0001
**Preoperative diagnosis**								
Advanced PDR (FVM and VH)	113,284.7 ± 5957.1 (89.7)		29,310.8 ± 883.3 (100.3)		15.4 ± 0.7 (94.3)		1.93 ± 0.07 (99.8)	
Rhegmatogenous Retinal Detachment	85,907.2 ± 2597.1 (81.9)	0.12	28,714.9 ± 375.1 (86.7)	0.003	16.5 ± 0.5 (91.2)	0.03	1.72 ± 0.08 (83.9)	0.004
Vitreomacular interface diseases (ERM/MH/VMT)	71,862.7 ± 1409.6 (76.4)		15,182.4 ± 532.1 (50.4)		9.9 ± 1.1 (56.0)		1.64 ± 0.08 (78.8)	
Endophthalmitis	114,542.9 ± 3973.5 (87.8)		64,800.0 ± 8356 (127.6)		17.0 ± 1.2 (90.0)		6.49 ± 2.6 (156.0)	
Dropped IOL or crystalline lens	211,751.8 ± 8758.2 (119.5)		26,763.1 ± 739 (94.4)		19.2 ± 1.1 (114.4)		1.46 ± 0.1 (64.9)	
**Associated cataract surgery with the primary PPV**	119,650.5 ± 7850 (94.2)	0.22	30,288.1 ± 750 (88.8)	0.67	16.3 ± 1.4 (91.7)	0.89	2.02 ± 0.2 (89.6)	0.78
**Duration from presentation for surgery (weeks)**	27.5 ± 153	0.95	−46.1 ± 72	0.53	−0.02 ± 0.03	0.55	0.001 ± 0.003	0.98
**Type of vitreous tamponade**								
Silicone oil	110,720.2 ± 4511 (88.2)		30,477.3 ± 901 (92.3)		16.4 ± 1.1 (93.7)		1.94 ± 0.2 (88.8)	
Gas	78,956.4 ± 5142 (78.3)	0.15	24,710.7 ± 413 (83.2)	0.41	13.9 ± 0.8 (82.2)	0.38	1.82 ± 0.1 (88.6)	0.51
Air	137,148.1 ± 7892 (100.5)		28,701.3 ± 564 (97.9)		15.8 ± 0.9 (95.9)		1.99 ± 0.1 (99.7)	
**Preoperative glaucoma**	176,623.6 ± 6235 (109.5)	0.018	33,336.9 ± 189 (105.4)	0.13	18.9 ± 1.6 (111.2)	0.034	1.77 ± 0.09 (92.4)	0.88
**Postoperative AG use**	93,821.2 ± 2289.1 (85.4)	0.82	29,030.9 ± 717.5 (91.9)	0.77	16.3 ± 1.3 (92.3)	0.55	1.84 ± 0.1 (90.4)	0.89
**HBA1c (%)**	3080.1 ± 663	0.39	130.7 ± 72	0.85	0.11 ± 0.2	0.72	−0.03 ± 0.04	0.55

**Abbreviations:** DM: diabetes mellitus; PDR: proliferative diabetic retinopathy; VH: vitreous hemorrhage; ERM: epiretinal membrane; VMT: vitreomacular traction; MH: macular hole; AG: anti-glaucoma; PPV: pars plana vitrectomy; SEM: standard error of mean; TIMP: tissue inhibitor of metalloproteinase; MMP: matrix metalloproteinase.

**Table 5:** Factors affecting the level of each biomarker separately in patients with RRD only.

Variables	Mean ± SEM (Mean Rank) or B Regression Coefficient ± SEM					
	TIMP1 (pg/mL)	*p*-Value	TIMP2 (pg/mg)	*p*-Value	MMP2 (ng/mL)	*p*-Value
**Age**	555.4 ± 84.7	0.51	99.1 ± 105.1	0.63	−0.06 ± 0.9	0.95
**Gender**						
Male	77,246.4 ± 1905 (31.5)	0.98	29,834.5 ± 507 (32.3)	0.64	16.4 ± 1.5 (32.1)	0.53
Female	108,835.6 ± 1472 (31.4)		25,792.9 ± 260 (34.8)		16.6 ± 1.1 (35.4)	
**Ocular parameters**						
Axial length (mm)	−1364.7 ± 170	0.74	1640.3 ± 700.7	0.34	0.58 ± 0.79	0.47
**Co-morbidities**						
Diabetes mellitus	95,311.0 ± 6368 (34.3)	0.49	21,388.9 ± 890 (28.2)	0.26	13.2 ± 2.1 (28.1)	0.25
Hypertension	67,560.1 ± 3084 (29.5)	0.52	22,916.8 ± 268 (32.0)	0.77	14.8 ± 1.7 (31.9)	0.73
**Macula status**						
Macula on	69,577.4 ± 2300 (24.1)	0.02	20,661.4 ± 693 (27.9)	0.11	12.4 ± 1.7 (25.5)	0.01
Macula off	94,272.4 ± 2411 (35.3)		33,125.2 ± 904 (35.8)		18.7 ± 2.1 (37.1)	
**Duration of RRD (days)**	−179.4 ± 242	0.88	6.7 ± 95.4	0.98	−0.01 ± 0.2	0.99
**Location of the tear**						
Within the superior half	61,062.1 ± 983 (28.5)	0.08	27,370.3 ± 251 (32.0)	0.59	15.6 ± 2.0 (31.3)	0.36
Within the inferior half	128,037.9 ± 1813 (36.6)		31,012.0 ± 639 (34.7)		17.9 ± 2.4 (35.8)	
**Number of involved quadrants**						
One or two quadrants	61,176.6 ± 703 (23.6)	0.0001	20,702.3 ± 855 (26.2)	0.001	12.1 ± 1.3 (25.4)	0.001
Three or four quadrants	115,939.1 ± 5097 (41.1)		39,303.6 ± 406 (41.9)		22.2 ± 2.8 (43.0)	
**Associated vitreous hemorrhage**	33,513.1 ± 822		20,942.9 ± 513		11.2 ± 4.2	
**Grade of PVR**						
No PVR	47,373.2 ± 379 (24.2)	0.0001	20,755.9 ± 323 (25.8)	0.0001	11.6 ± 1.2 (25.0)	0.001
Grade A or B	72,270.2 ± 422 (29.8)		26,738.8 ± 789 (32.2)		15.2 ± 1.8 (32.5)	
Grade C or D	19,6058.5 ± 858 (51.2)		51,806.7 ± 478 (52.5)		30.7 ± 4.9 (54.1)	
**Preoperative glaucoma**	236,404.3 ± 2597 (44.5)	0.14	26,801.7 ± 593 (36.8)	0.69	18.5 ± 5.3 (36.3)	0.72
**Postoperative AG use**	66,365.7 ± 2253 (29.9)	0.48	27,633.7 ± 745 (31.6)	0.32	16.0 ± 2.3 (31.0)	0.39
**Homocystein**	720.2 ± 79	0.42	354.2 ± 23	0.29	0.13 ± 0.12	0.31

**Abbreviations:** RRD: rhegmatogenous retinal detachment; AG: anti-glaucoma; PVR: proliferative vitreoretinopathy; SEM: standard error of mean; TIMP: tissue inhibitor of metalloproteinase; MMP: matrix metalloproteinase.

## Data Availability

The datasets used and/or analyzed during the current study are presented in tables and text.
